# Listening habits and subjective effects of background music in young adults with and without ADHD

**DOI:** 10.3389/fpsyg.2024.1508181

**Published:** 2025-01-22

**Authors:** Kelly-Ann Lachance, Pénélope Pelland-Goulet, Nathalie Gosselin

**Affiliations:** ^1^International Laboratory for Brain, Music and Sound Research (BRAMS), Center for Research on Brain, Language and Music (CRBLM), Laboratory for Music, Emotions and Cognition Research (MUSEC), Interdisciplinary Research Center on Brain and Learning (CIRCA), CerebrUM Research Center, Department of Psychology, University of Montreal, Montreal, QC, Canada; ^2^Alpha Neuro Center, Montmorency College, Montreal, QC, Canada; ^3^Neurocognition Vision Laboratory, University of Montreal, Montreal, QC, Canada

**Keywords:** background music, music listening habits, subjective effect, cognition, arousal, musical emotions, ADHD, attention

## Abstract

Adults listen to an average of 20.7 hours of music per week, according to a study conducted across 26 countries. Numerous studies indicate that listening to music can have beneficial effects on cognitive performance and emotional well-being. Music listening habits may vary depending on individual needs and listening contexts. However, a limited number of studies have specifically examined the patterns of background music usage during various more or less cognitive activities, especially among individuals with attentional difficulties related to ADHD. This study primarily aimed to compare music listening habits during daily activities that are more and less cognitive (e.g., studying, problem-solving versus cleaning, engaging in sports) between neurotypical young adults and those screened for ADHD (respondents who were identified as likely having ADHD based on the number of self-reported symptoms). To achieve this, 434 young adults aged 17 to 30 responded to an online survey. The results indicate that certain listening habits differ significantly between the neurotypical and ADHD-screened groups. The ADHD-screened group reports significantly more background music listening during less cognitive activities and while studying, compared to the neurotypical group. The results also reveal a difference in the proportion of individuals preferring stimulating music between the groups: ADHD-screened individuals report significantly more frequent listening to stimulating music, regardless of the activity type (more or less cognitive). Other aspects of music listening are common to both groups. Regardless of the group, more respondents reported preferring to listen to relaxing, instrumental, familiar and self-chosen music during more cognitive activities, whereas for less cognitive activities, more individuals mentioned preferring to listen to music that is stimulating, with lyrics, familiar and self-chosen. Overall, the results confirm that most young adults listen to music during their daily activities and perceive positive effects from this listening.

## 1 Introduction

Music is omnipresent in our daily lives (Hu et al., 2021; International Federation of the Phonographic Industry, 2023; North et al., 2004), serving diverse functions such as emotion regulation, concentration enhancement, and providing background engagement during other activities (Chamorro-Premuzic and Furnham, 2007; Goltz and Sadakata, 2021; Hu et al., 2021; Kotsopoulou and Hallam, 2010; Mas-Herrero et al., 2013; Schäfer et al., 2013). While listening to music can be the primary focus of some activities, background music (BM) refers to music that is not the main focus of attention but is present while the listener engages in another primary activity (Lonsdale and North, 2011; Schellenberg and Weiss, 2013). A UK study found that among 18 to 29 year-olds, BM was listened to during cognitive activities (tasks requiring high cognitive load) such as studying and writing up to 71% of the time (Greasley and Lamont, 2011). Researchers have explored both the musical characteristics and emotions evoked by music (e.g., stimulating vs. relaxing music) that may differently influence performance on cognitive tasks (Gonzalez and Aiello, 2019; Schellenberg and Weiss, 2013). According to previous studies, listening to music can exert both detrimental and beneficial effects on attention (Christopher and Shelton, 2017; Mendes et al., 2021). For example, some researchers demonstrated that individuals who are better at controlling their attention are less distracted by music during certain cognitive tasks (Christopher and Shelton, 2017; Goltz and Sadakata, 2021; Pelletier et al., 2016; Salmi et al., 2020). BM is also commonly associated with various benefits such as emotion regulation, which can positively influence performance (Juslin and Västfjäll, 2008). However, studies show heterogeneity in its objective effects on various experimentally measured cognitive activities (Kämpfe et al., 2010). For instance, while some studies report that BM may enhance concentration and memory in neurotypical individuals (Gonzalez and Aiello, 2019; Kotsopoulou and Hallam, 2010; Cournoyer Lemaire, 2019), others indicate that it may disrupt attentional control, memory, and reading (Cloutier et al., 2020; Kämpfe et al., 2010; Schellenberg and Weiss, 2013). These heterogeneous results could be partly explained by diverse methodologies and different perceptions each individual has toward the music they listen to (Cheah et al., 2022; Gonzalez and Aiello, 2019; Kiss and Linnell, 2023). Thus, people may adapt their music listening habits based on their emotional needs (Thompson et al., 2001) and the cognitive resources required to perform a task, considering (1) its difficulty level, and (2) the musical characteristics used (Goltz and Sadakata, 2021). Indeed, several studies investigated the impacts of BM listening during more cognitive (see Cheah et al., 2022 for a review) and less cognitive activities (Clark et al., 2022). Recent studies also compared uses and impacts of BM across tasks difficulties (Kiss and Linnell, 2023; Goltz and Sadakata, 2021) Nevertheless, research on listening habits and their subjective effects remains limited to date, particularly in individuals with atypical attention, such as individuals with Attention Deficit with/without Hyperactivity Disorder (ADHD). The objective of this study is to detail and compare BM listening habits during tasks with varying cognitive demands between young adults with a neurotypical profile and those who screen positively for ADHD, as well as to examine BM’s subjective impact on cognitive and emotional functioning during more demanding tasks. By documenting both the listening habits and the perceived subjective effects of BM in these two populations, this study seeks to enhance our understanding of BM’s potential as a practical tool for attention-related conditions.

ADHD is a neurodevelopmental disorder characterized by symptoms of inattention or hyperactivity and impulsivity (American Psychiatric Association, 2013). It typically manifests during early childhood, and some symptoms persist into adulthood in 50 to 65% of cases (Agnew-Blais et al., 2016; Biederman et al., 2006; Caye et al., 2016). ADHD is classified into three subtypes: inattentive, hyperactive–impulsive, and combined (American Psychiatric Association, 2013). Among adults, the inattentive subtype (characterized by difficulties in sustaining attention, being organized, and following instructions, without necessarily displaying restless or impulsive behaviors) is often predominant (Ustun et al., 2017). Adults with ADHD commonly experience comorbid conditions or associated symptoms such as anxiety and depression, especially in untreated cases (Barkley, 2008; Riboldi et al., 2022; Sahmurova et al., 2022). Studies focusing on ADHD populations should therefore consider these additional symptoms. The prevalence of ADHD among adults worldwide is 2.6% (Simon et al., 2009; Song et al., 2021). In Canada, the prevalence varies from 2.9% (Hesson and Fowler, 2018) to 7.3% (Morkem et al., 2020). In addition to the more challenging transition to adulthood experienced by individuals with ADHD due to the functional impacts of the disorder (Lagacé-Leblanc et al., 2020), ADHD can affect certain neurophysiological mechanisms and systems, such as the dopaminergic system.

The dopaminergic system (also known as the reward system) refers to the brain system responsible for dopamine production, transmission, and regulation, critical for functions such as motivation, reward processing, and other key neurological processes (Nestler and Carlezon, 2006; Wise, 2004). An attenuated and dysfunctional dopaminergic system is involved in ADHD (Volkow et al., 2009; Wu et al., 2012). This dysfunction aligns with the Moderate Brain Arousal model (MBA), which suggests that individuals with ADHD require higher brain arousal levels than neurotypicals to reach a moderate (optimal) level in the dopaminergic system and improve performance, while too little or too much brain arousal impairs performance (Batho et al., 2020; Sikström and Söderlund, 2007; Strauß et al., 2018). This regulation of brain arousal levels was initially described by Zentall (1975), who highlighted arousal regulation particularities in people with ADHD. The author proposed that cortical hypoactivation contributes to ADHD symptoms (e.g., inattention, hyperactivity), which can be alleviated by medications like methylphenidate, a central nervous system stimulant (Volkow et al., 2009; Zimmermann et al., 2019). However, the literature suggests another, non-pharmaceutical, means of controlling arousal levels: listening to music (Dillman Carpentier and Potter, 2007; Schäfer et al., 2013; Salimpoor et al., 2011; Woods et al., 2024). Indeed, a recent study showed that music which contains certain characteristics can have positive effects on sustained attention performance in individuals with high ADHD symptoms (Woods et al., 2024). Furthermore, several studies (Blood and Zatorre, 2001; Ferreri et al., 2019; Salimpoor et al., 2011, 2015; Zatorre and Salimpoor, 2013), have demonstrated the activation of the reward system, specifically the release of dopamine (i.e., the pleasure neurotransmitter) by the nucleus accumbens during music listening. Moreover, other researchers have reported that in silent conditions, adults with ADHD seem to have more difficulties with arousal regulation due to hypo-arousal (where low arousal levels lead to discomfort; MBA: Sikström and Söderlund, 2007) compared to neurotypicals (Zimmermann et al., 2019). They have also indicated that listening to music improves their mood (emotional arousal) (Zimmermann et al., 2019).

Indeed, music is recognized for its ability to modulate and induce emotions (Hunter and Schellenberg, 2010; Juslin et al., 2010; Lonsdale and North, 2011; Schäfer et al., 2013; Schellenberg and Weiss, 2013) and subsequently impact cognitive performance (Papinczak et al., 2015; Schäfer et al., 2013). This relationship is supported by the Mood Arousal Theory (Thompson et al., 2001). Music influences perceived emotions through its valence (pleasant or unpleasant) and the activation level it can induce (relaxing or stimulating) (Vieillard et al., 2008), two dimensions of emotions frequently used in research (Eerola and Vuoskoski, 2011; Lang et al., 1998; Vieillard et al., 2008). Pleasant emotions and stimulation generated by music seem to enhance cognitive performance (Husain et al., 2002; Oaksford et al., 1996; Thompson et al., 2001). Furthermore, the level of available attentional resources varies depending on the level of arousal (Thompson et al., 2001). According to the Cognitive Capacity Hypothesis (CCH, Kahneman, 1973), tasks requiring higher cognitive load demand more cognitive resources, which can negatively affect performance when demand exceeds available attentional resources (Chou, 2010; Kang and Lakshmanan, 2017; Kotsopoulou and Hallam, 2010). BM can thus influence cognitive performance, especially during complex tasks, where high arousal levels can become distracting and deteriorate performance (Cassidy and MacDonald, 2007; Smith and Morris, 1977). Furthermore, during certain cognitive tasks, multiple auditory stimuli, including music, can sometimes be problematic for individuals with ADHD due to their greater vulnerability to distractions (Pelletier et al., 2016; Salmi et al., 2020). However, its effect may vary depending on the nature of the task and the attentional reserve of each individual (Kang and Lakshmanan, 2017). Thus, the amount of attention required during multitasking depends on the demand of each activity. For example, according to Hallam (2012) and Kiger (1989), stimulating music can enhance performance on simple tasks by increasing arousal, but may impair performance on complex tasks if arousal becomes excessive. In conclusion, the Mood Arousal Theory and the Cognitive Capacity Hypothesis offer plausible explanations for BM’s subjective effects on emotions and cognitive performance (Goltz and Sadakata, 2021). Multiple studies found evidence that music is often perceived by individuals as a means to enhance cognitive performance and regulate emotions, particularly improving mood during cognitive activities (Chamorro-Premuzic and Furnham, 2007; Goltz and Sadakata, 2021; Hu et al., 2021; Mas-Herrero et al., 2013; Papinczak et al., 2015; Schäfer et al., 2013). For instance, in a study by Kotsopoulou and Hallam (2010), participants reported that listening to BM during cognitive tasks reduced boredom, aided relaxation, and enhanced concentration. However, discrepancies exist between the perceived subjective effects and the objective effects of BM evaluated through experimental tasks, such as those concerning reading (Alley and Greene, 2008; Johansson et al., 2011; Juslin and Laukka, 2004; Kotsopoulou and Hallam, 2010). Therefore, it is relevant to understand behaviors associated with BM use and to explore its effects on daily listening habits.

Personal use of BM may vary depending on the difficulty of the task at hand. In addition, musical preferences for various activities may affect task performance differently, either positively or negatively (Chamorro-Premuzic and Furnham, 2007; Johansson et al., 2011; Towell, 1999). People generally report preferring instrumental, relaxing, and classical music as background for cognitive tasks (Goltz and Sadakata, 2021). However, the use of familiar music might have a detrimental effect on cognitive performance, as familiarity with the music could potentially distract from the main task (Zhang et al., 2009). Nonetheless, some studies suggest that familiar music is more pleasurable than unfamiliar music and can ultimately enhance performance (Freitas et al., 2018; Zajonc, 1968). Additionally, being able to choose music for oneself seems to promote learning more effectively than non-chosen music by better meeting individual activation needs, thus providing an optimal level of stimulation for performance (Anyanwu et al., 2016). Therefore, different musical characteristics should be prioritized when performing cognitive activities (Schellenberg and Weiss, 2013). People who report positive effects of BM listening, such as improved concentration or mood, tend to listen to BM more frequently than those who report negative or no notable effects on various tasks (Goltz and Sadakata, 2021; Kotsopoulou and Hallam, 2010). Furthermore, a limited number of studies have specifically examined BM listening habits for different activities, such as more (high cognitive load) or less (low cognitive load) cognitive in daily life (Goltz and Sadakata, 2021; Kiss and Linnell, 2023; Mendes et al., 2021). Moreover, preferred musical habits in daily life have been explored only minimally, as most research focuses on cognitive abilities in the presence of BM using experimental tasks in laboratory settings. This highlights the importance of exploring BM listening habits and preferred musical characteristics during more cognitive (e.g., reading, writing) and less cognitive (e.g., cleaning, engaging in sports) activities.

According to the literature, musical experience can have both positive and negative effects on cognitive performance when listening to BM (Drai-Zerbib and Baccino, 2017; Herholz and Zatorre, 2012). For instance, due to their daily practice of tasks requiring a variety of cognitive resources and functions, musicians tend to have better attention while working with BM compared to non-musicians (Drai-Zerbib and Baccino, 2017; Rodrigues et al., 2013; Wickens, 2002; Wu and Shih, 2021). However, other studies have shown that listening to BM can represent an additional cognitive load for musicians, as they perceive the music (e.g., musical arrangements) differently than non-musicians (Patston and Tippett, 2011; Schellenberg and Weiss, 2013; Yoo et al., 2022). Given the influence of musical training on performance in various cognitive tasks, studies must account for musical training in their analyses.

In summary, very little research exists on self-reported listening habits during activities of varying complexity (Kiss and Linnell, 2023). Moreover, a recent literature review by Mendes et al. (2021) suggests that listening to music improves attention performance and that the effect of music is influenced by the participants’ mood, arousal/state, and musical characteristics (e.g., the presence or absence of lyrics). However, these authors highlight important limitations in the literature: (1) in several studies, music is selected by the researchers; (2) some studies did not describe their musical selection process; and (3) very few studies considered the participants’ musical preferences. Evidently, more data is needed to determine the potential applications of music in clinical conditions that affect attention (Mendes et al., 2021).

The main goal of the study was to document BM listening habits and its perceived impact on cognition and emotion using an online survey, and to compare these observations between two groups of young adults; those with a neurotypical profile and those who screened positively for ADHD.

More specifically, the primary aim of this study was to detail and compare BM listening habits between young adults with a neurotypical profile and those who screened positively for ADHD. To achieve this, the number of hours per week spent listening to music, both as a primary activity and as a secondary activity, and during two types of activities [more cognitive activities (e.g., writing) and less cognitive activities (e.g., cleaning)] was documented. Musical characteristics (e.g., with or without lyrics) and musical styles were also compared between groups and the two types of activities (more or less cognitive).

No specific hypotheses were made regarding the comparison of listening habits between groups.

The secondary aim of this study was to detail and compare the subjective effects of BM on cognitive and emotional functioning, within (cognitive vs. emotional functioning) and between both groups (neurotypical vs. ADHD-screened), during more cognitively demanding activities. We focused exclusively on the effects of BM during more cognitive activities to more precisely observe its impacts on cognitive and emotional functioning. To do so, exploratory factor analysis was used to identify potential subcategories (factors) of subjective effects of BM. Additionally, other variables that could influence listening habits and subjective effects of BM were taken into account, such as the number of years of musical training and the depressive and anxious state of respondents.

Considering the literature, the questionnaire was created with two elements in mind: the subjective effects of BM listening during more cognitive tasks on (a) cognitive functioning and (b) emotional functioning. Consequently, two main factors were expected to emerge from these items: cognitive functioning (e.g., “My concentration is enhanced thanks to background music.”) and emotional functioning (e.g., “Background music improves my mood.”). For these factors, two hypotheses were formulated. First, in accordance with the Cognitive Capacity Hypothesis [which suggests that attentional resources are limited and must be shared among different activities, Kahneman (1973)], and considering that the ADHD-screened group should exhibit more symptoms of inattention than the neurotypical group, it was predicted that the ADHD-screened group would perceive more negative impacts of BM on their cognitive functioning than the neurotypical group. Second, due to the greater sensitivity and need for activation in ADHD (Moderate Brain Arousal model: Sikström and Söderlund, 2007), it was predicted that the ADHD-screened group would perceive a greater positive effect of BM on emotional functioning than those in the neurotypical group (Mood Arousal Theory: Thompson et al., 2001).

## 2 Materials and methods

### 2.1 Participants

**Figure 1:**
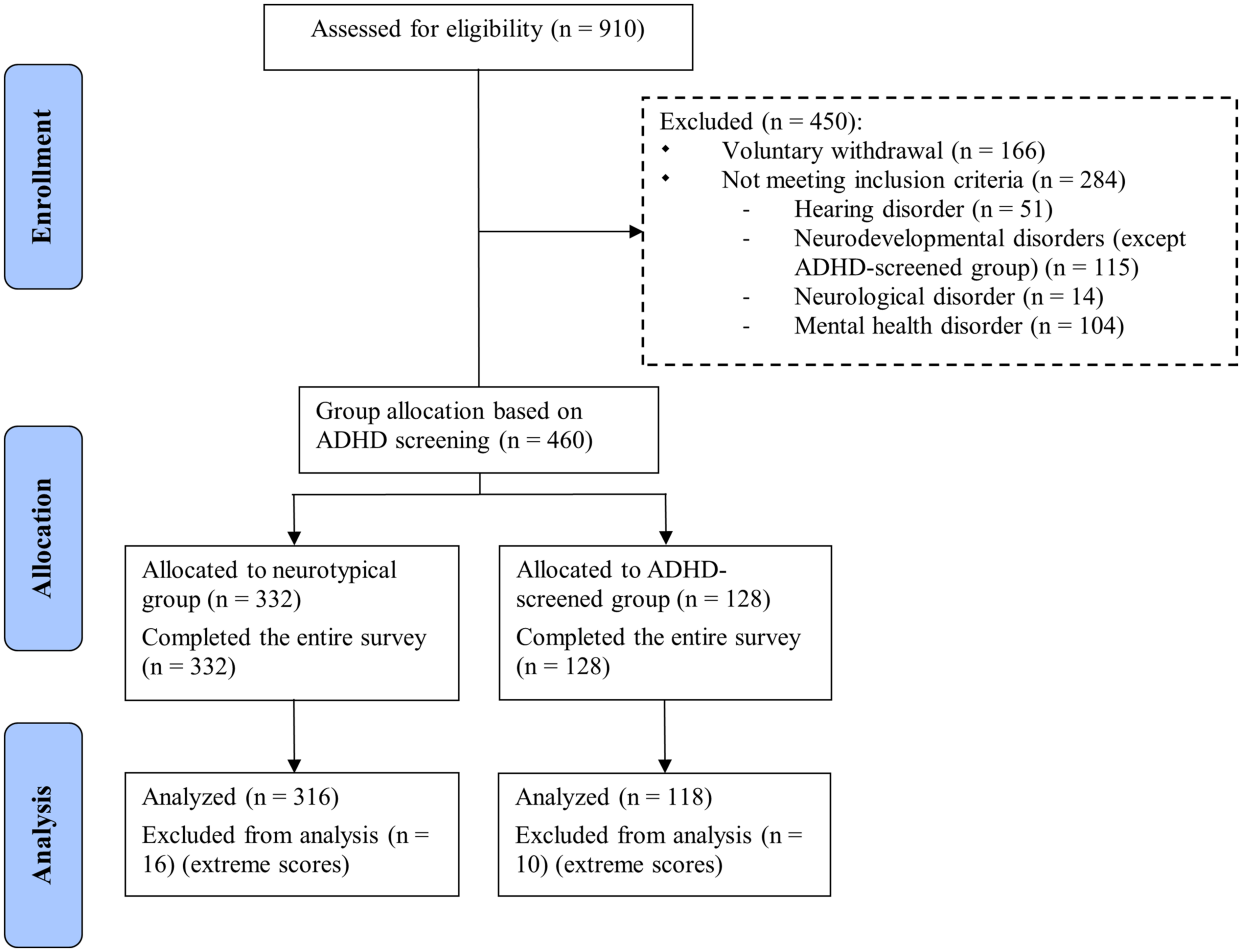
Participation flow diagram (July 2022 to October 2023). This diagram was inspired by the CONSORT diagram, which aims to transparently report data (Schulz et al., 2010). The scores were considered extreme (26) when z-score = ± 3.29 (Tabachnick and Fidell, 2013). The final sample size was *n* = 434 participants. Using the adult ADHD Self-Report Screening Scale for DSM-5 (ASRS-5), the sample was divided into two groups, one where participants were neurotypical, and the other one with participants who screened positively for ADHD.

A total of 910 individuals aged 17 to 30 years old and proficient in written French completed the survey, and 434 were retained for analysis (see Figure 1 for details on participation flow). Indeed, 476 participants were excluded for the following reasons: voluntary withdrawal (e.g., closing the form before submitting it), hearing impairment, neurodevelopmental disorder (excluding ADHD for the ADHD-screened group), neurological disorder, mental health disorder, and extreme scores on descriptive and sociodemographic variables. Among the final sample of 434 respondents (Mean Age = 23.29, SD = 3.76), 263 identified as women (60.6% of the sample), 157 as men (36.2%), and 14 as gender diverse individuals (3.2%). Most respondents reported French (52.8%) or English (33.9%) as their native language and were of North American (56.5%), European (14.7%), or South American or Central American origin (14.5%). All participants provided informed consent. This study was approved by the Research Ethics Committee in Education and Psychology of the University of Montreal.

### 2.2 Measures

#### 2.2.1 Adult ADHD self-report screening scale for DSM-5 (ASRS-5)

The French version of the Adult ADHD Self-Report Screening Scale for DSM-5 (ASRS-5: Ustun et al., 2017; Baggio et al., 2021) was used to screen for symptoms related to ADHD. This scale, also employed in clinical settings (Baggio et al., 2021; Harrison and Edwards, 2023; Timmermans et al., 2020; Weibel et al., 2018), comprises six items assessing symptoms associated with ADHD. For each item, respondents indicated how they felt or behaved over the past 6 months using a five-point Likert scale ranging from never (0 points) to very often (4 points), yielding a maximum score of 24. Studies have recommended different screening thresholds for various ADHD subgroups based on symptomatology, highlighting differences in targeted study populations and reasons for ASRS-5 usage (Baggio et al., 2021; Bastiaens and Galus, 2018; Dubois et al., 2023; Genç et al., 2021). For this study, the threshold of ≥12/24 for positive ADHD screening was chosen, as it provides good validity (Baggio et al., 2021; Somma et al., 2021), specificity (85.5%) and sensitivity (89.4%) in identifying potential cases of predominantly inattentive presentation ADHD, which is often prevalent in adults (Baggio et al., 2021; Ustun et al., 2017). Thus, respondents scoring at or above the threshold (≥ 12/24) on the ASRS-5 were assigned to the ADHD-screened group, while those scoring below were assigned to the neurotypical group.

#### 2.2.2 Listening habits for background music

The average number of hours spent listening to music per week, both as a primary and secondary activity, was collected. Music listening habits during the performance of more cognitive activities and less cognitive activities (e.g., reading and writing versus cleaning and engaging in sports) were gathered using 12 questions inspired by those previously used in the literature (Chamorro-Premuzic and Furnham, 2007; Goltz and Sadakata, 2021; Greasley and Lamont, 2011; Hu et al., 2021; Kotsopoulou and Hallam, 2010) (see Table 1 for questionnaire items and Supplementary Appendix A for the complete questionnaire). Listening habits for BM based on activity types were measured using a Likert scale ranging from 1 (never) to 7 (very often). Additionally, musical characteristics and dimensions of emotions evoked by music such as activation (relaxing or stimulating), presence or absence of lyrics, familiarity, chosen or not by the respondent, as well as preferred musical styles during more cognitive and less cognitive activities, were collected. Each of these characteristics was documented using four response choices (e.g., for activation: relaxing, stimulating, no preference, and not applicable; see Table 1).

**Table 1:** Background music listening habits questionnaire.

How many hours per week (on average) do you listen to music as a primary activity? ☐ 0 ☐ 1 ☐ 2 ☐ 3 … ☐ 168
How many hours per week (on average) do you listen to music as a secondary activity? ☐ 0 ☐ 1 ☐ 2 ☐ 3 … ☐ 168
Using the scale below, ranging from 1 (Never) to 7 (Very often), indicate how frequently you listen to music during the following MORE cognitive activities. While studying ☐ 0 ☐ 1 ☐ 2 ☐ 3 ☐ 4 ☐ 5 ☐ 6 ☐ 7 While memorizing ☐ 0 ☐ 1 ☐ 2 ☐ 3 ☐ 4 ☐ 5 ☐ 6 ☐ 7 For problems-solving or calculations ☐ 0 ☐ 1 ☐ 2 ☐ 3 ☐ 4 ☐ 5 ☐ 6 ☐ 7 While reading ☐ 0 ☐ 1 ☐ 2 ☐ 3 ☐ 4 ☐ 5 ☐ 6 ☐ 7 While writing ☐ 0 ☐ 1 ☐ 2 ☐ 3 ☐ 4 ☐ 5 ☐ 6 ☐ 7 While learning (e.g., new language) ☐ 0 ☐ 1 ☐ 2 ☐ 3 ☐ 4 ☐ 5 ☐ 6 ☐ 7 For engaging in logic puzzles (e.g., Sudoku) ☐ 0 ☐ 1 ☐ 2 ☐ 3 ☐ 4 ☐ 5 ☐ 6 ☐ 7
Specify which style(s) of music you listen to when performing these MORE cognitive activities: ☐ None ☐ Alternative or Indie ☐ Jazz or Blues ☐ Classical or Opera ☐ Country, Western ☐ Dance, Techno, or Electronic ☐ Latin Music ☐ Ballad ☐ Folk ☐ Gospel ☐ Metal or Punk ☐ Soul ☐ Popular Music from Your Culture ☐ Pop ☐ World Music ☐ Rap or Hip-Hop ☐ Reggae ☐ Traditional Music from Your Culture ☐ Rock ☐ R&B ☐ Music from Films, TV Shows, Video Games ☐ Other: ____
When you listen to music while performing your MORE cognitive activities, do you prefer the music to be: ☐ Does not apply ☐ Relaxing ☐ Stimulating ☐ No preference ☐ Does not apply ☐ Without lyrics ☐ With lyrics ☐ No preference ☐ Does not apply ☐ Familiar ☐ Unfamiliar ☐ No preference ☐ Does not apply ☐ Chosen by you ☐ The choice of music does not matter ☐ No preference
Using the scale below, ranging from 1 (Never) to 7 (Very often), indicate how frequently you listen to music during the following LESS cognitive activities. While cleaning ☐ 0 ☐ 1 ☐ 2 ☐ 3 ☐ 4 ☐ 5 ☐ 6 ☐ 7 During commuting/public transportation ☐ 0 ☐ 1 ☐ 2 ☐ 3 ☐ 4 ☐ 5 ☐ 6 ☐ 7 While cooking at home ☐ 0 ☐ 1 ☐ 2 ☐ 3 ☐ 4 ☐ 5 ☐ 6 ☐ 7 While engaging in sports ☐ 0 ☐ 1 ☐ 2 ☐ 3 ☐ 4 ☐ 5 ☐ 6 ☐ 7
Specify which style(s) of music you listen to when performing these LESS cognitive activities: ☐ None ☐ Alternative or Indie ☐ Jazz or Blues ☐ Classical or Opera ☐ Country, Western ☐ Dance, Techno, or Electronic ☐ Latin Music ☐ Ballad ☐ Folk ☐ Gospel ☐ Metal or Punk ☐ Soul ☐ Popular Music from Your Culture ☐ Pop ☐ World Music ☐ Rap or Hip-Hop ☐ Reggae ☐ Traditional Music from Your Culture ☐ Rock ☐ R&B ☐ Music from Films, TV Shows, Video Games ☐ Other: _______
When you listen to music while performing your LESS cognitive activities, do you prefer the music to be: ☐ Does not apply ☐ Relaxing ☐ Stimulating ☐ No preference ☐ Does not apply ☐ Without lyrics ☐ With lyrics ☐ No preference ☐ Does not apply ☐ Familiar ☐ Unfamiliar ☐ No preference ☐ Does not apply ☐ Chosen by you ☐ The choice of music does not matter ☐ No preference

The complete version of this questionnaire can be found in Supplementary Appendix A.

#### 2.2.3 The subjective effects of background music on cognitive functioning and emotional functioning

The perception of the effect of music listened to during the performance of more cognitive activities was initially documented with 27 statements where respondents rated their level of agreement on a Likert scale from 1 indicating total disagreement to 7 indicating total agreement (see Table 2 for questionnaire items and Supplementary Appendix B for the complete questionnaire). We focused exclusively on the effects of BM during more cognitive activities to more precisely observe its impacts on cognitive and emotional functioning. The statements were constructed based on existing questionnaires in the literature, focusing on the perception of music’s effect on concentration, as well as on identifying the uses and functions of music (e.g., music helps me calm down) (Chamorro-Premuzic and Furnham, 2007; Kotsopoulou and Hallam, 2010; Schäfer et al., 2013).

**Table 2:** Background music effects on performance and emotions questionnaire.

This part of the survey aims to explore the effect of background music on your performance in daily activities of a “cognitive nature” (e.g., studying, memorizing, reading, writing). Referring to the scale below, please respond to the following statements by selecting the corresponding number, where 1 = Strongly disagree and 7 = Strongly agree. Make sure to respond to all statements as accurately as possible. Background music refers to listening to music as a secondary activity while you perform a primary task (e.g., listening to music while reading).						
1 = Strongly disagree	2 = Disagree	3 = Slightly disagree	4 = Neutral	5 = Slightly agree	6 = Agree	7 = Strongly agree
**1. Background music allows me to concentrate better.**						
2. Background music helps me overcome boredom when engaging in cognitive activities.						
**3. Background music helps to make me more alert.**						
**4. Background music brings me a sense of joy.**						
**5. My performance is better when I engage in cognitive activities with music.**						
**6. Background music reduces my stress.**						
7. Background music makes cognitive tasks less boring.						
**8. Background music improves my mood.**						
9. I believe that music interferes with my concentration.						
**10. Background music acts as a good stimulant for performing cognitive activities.**						
11. Background music reduces my ability to memorize information.						
**12. Background music reduces my boredom feelings.**						
13. Background music increases my stress while engaging in cognitive activities.						
14. Background music distracts me from my primary task.						
**15. Background music makes me happy.**						
16. Background music helps me relax when engaging in cognitive activities.						
17. Background music disrupts my performance on cognitive tasks.						
18. Background music evokes sense of excitation in me.						
**19. My concentration is enhanced thanks to background music.**						
20. I get stressed when I engage in cognitive activities with background music.						
**21. Background music makes my mood less negative.**						
**22. Background music 23. positively influences my performance on cognitive tasks.**						
**23. Background music heightens my senses.**						
**24. Background music improves my concentration during cognitive activities.**						
25. Background music reduces my productivity.						
26. Background music takes up too much of my attention while I am doing cognitive activities.						
**27. Background music helps me memorize new information.**						

Statements in bold indicate those retained after the EFA. The complete version of this questionnaire can be found in Supplementary Appendix B.

#### 2.2.4 Profile of mood state (POMS) and profile of mood state adolescents (POMS-A)

For respondents aged 18 and older, the depression subscale (three items: discouraged, sad, and hopeless) and the tension-anxiety subscale (three items: on edge, nervous, and anxious) from the abbreviated version of the POMS were utilized (Cranford et al., 2006; Fillion and Gagnon, 1999; Shacham, 1983). Respondents indicated how well each individual item represented their state over the past week using Likert scales from 0 (not at all) to 4 (extremely). The POMS is generally used for individuals aged 18 and older. To accurately survey the population of this study (adults aged 17 to 30 years), the POMS-A was also used for those aged 17. Participants aged 17 completed a similar version of the POMS for adolescents, which includes four items for the depression subscale (discouraged, depressed, miserable, and unhappy) and four items for the tension-anxiety subscale (panicked, nervous, anxious, and worried) (POMS-A; Terry et al., 1999). Both subscales were computed using the average score of their respective items. For simplicity, both the POMS and the POMS-A are referred to as POMS in the text.

### 2.3 Procedure

Data collection was carried out from July 2022 to October 2023 using an online survey administered through *LimeSurvey* software (LimeSurvey GmbH, 2020; Version 3.28.52). The survey lasted 10 to 15 min and was distributed through email lists and social media platforms, among other channels (e.g., online forums, websites, and community groups). All participants first read and completed the consent form, then provided general information to describe the sample’s sociodemographic characteristics (e.g., gender identity, age, musical training) and to determine eligibility (e.g., absence of hearing impairments, absence of neurodevelopmental disorders). Subsequently, participants completed the ASRS-5, followed by questions about music listening habits and the subjective effects of BM, and concluded with the POMS subscales.

### 2.4 Data analysis

#### 2.4.1 Data integrity

There were no missing values identified since data from participants who did not submit their responses was not considered and since all items had to be answered to continue completing the online form. Non-parametric analyses were used when variables were not normally distributed, whereas parametric analyses were used when variables were normally distributed (i.e., skewness and kurtosis values of +/− 1) (Hair et al., 2010), with *α* = 0.05 for all analyses.

#### 2.4.2 Group characteristics (neurotypical and ADHD-screened)

Both groups (neurotypical, ADHD-screened) were compared on gender identity and completed level of education using Chi-square tests. Age, number of years of musical training, as well as the average scores of the POMS depression and tension-anxiety subscales were analyzed using one-way analysis of variance (ANOVA) with one factor (group) and two levels (neurotypical, ADHD-screened).

#### 2.4.3 Background music listening habits

Two independent samples Mann–Whitney U tests were conducted to compare neurotypical and ADHD-screened participants on the amount of time per week spent listening to BM as a primary and secondary activity. Additionally, for each group, music listening habits were compiled using means from a Likert scale ranging from 1 (never) to 7 (very often) for more cognitive activities (studying, memorizing, problem-solving, reading, writing, learning, and engaging in logic games) and less cognitive activities (cleaning, during commutes/public transportation, cooking at home, and engaging in sports). The activities were separated into “more cognitive” and “less cognitive” classes based on previous use of such distinctions (e.g., Kiss and Linnell, 2023). The average BM listening for the two types of activities between the neurotypical group and the ADHD-screened group was compared using a repeated measures ANOVA with a between-subjects factor group (neurotypical and ADHD-screened) and a within-subject factor activity type (more and less cognitive). More and less cognitive activity types were also analyzed separately using ANOVAs with a factor (group) at two levels (neurotypical, ADHD-screened). Multiple regressions were also used to control for potential confounding variables (years of musical training as well as mean total scores of the POMS depression and tension-anxiety subscales) when significant differences between the neurotypical group and the ADHD-screened group were observed. Additionally, Chi-square tests comparing both groups on the frequencies of respondents who selected each BM characteristic as their preferred characteristics (i.e., relaxing or stimulating, with or without lyrics, familiar or unfamiliar, and chosen or not by the respondent) and for preferred musical styles (e.g., Rock, Classical, Jazz, Hip Hop…), separately for more and less cognitive activities were conducted.

#### 2.4.4 Subjective effects of BM on cognitive functioning and emotional functioning

An exploratory factor analysis (EFA) was first conducted to explore the data structure of the 27 initial statements measuring the subjective effects of BM (Likert scale from 1 indicating total disagreement to 7 indicating total agreement). The EFA also enabled the verification of whether the two expected factors, subjective effects on cognitive functioning and subjective effects on emotional functioning, were identified. Factors resulting from the EFA were then analyzed using a one-way ANOVA to compare neurotypical and ADHD-screened groups based on the mean scores from the Likert scale for each factor. All statistical analyses were performed using IBM SPSS Statistics [version: 28.0.1.0 (142)].

## 3 Results

### 3.1 Comparison of characteristics between neurotypical and ADHD-screened groups

The final sample was made of 316 participants who did not reach the positive screening on the ASRS-5 (neurotypical group) and 118 participants who reached the threshold for positive screening on the ASRS-5 (ADHD-screened group).

The neurotypical and ADHD-screened groups were equivalent in terms of gender identity, age, completion of a pre-university or undergraduate program, and current post-secondary student status (see Table 3). However, a significantly higher frequency of adults completed a graduate university program in the neurotypical group compared to the ADHD-screened group. In addition, the neurotypical group had significantly more years of musical training than the ADHD-screened group. Finally, the ADHD-screened group scored significantly higher than the neurotypical group on average on the depression and tension-anxiety POMS subscales.

**Table 3:** Sociodemographic characteristics and depressive and anxious state across groups (Neurotypical, ADHD-screened).

	Neurotypical (*n* = 316)	ADHD-screened (*n* = 118)	*F*(df1, df2) --- *χ^2^* (*1, 434*)	*p-value*	η^2^ --- Cramer’s V
Gender Identity					
Women Men Other	190 (60.1%) 114 (36.1%) 12 (3.8%)	73 (61.9%) 43 (36.4%) 2 (1.7%)	0.109 0.005 1.217	0.742 0.944 0.270	0.016 0.003 0.053
Age	23.43 (3.74)	22.92 (3.79)	1.56 (1, 432)	0.212	0.004
Present post-secondary students					
Completed Level of Education Pre-university Undergraduate University Graduate University None	240 (75.9%) 115 (36.4%) 127 (40.2%) 74 (23.4%) 0 (0.0%)	87 (73.7%) 54 (45.8%) 51 (43.2%) 9 (7.6%) 4 (3.4%)	0.228 3.173 0.326 13.851 --	0.633 0.075 0.568 < 0.001 --	0.023 0.086 0.027 0.179 --
Years of Musical Training (*n* = 206)	7.90 (4.84)	5.66 (4.72)	7.47 (1, 204)	0.007	0.035
POMS					
Depression Tension-Anxiety	0.85 (0.74) 1.19 (0.85)	1.49 (0.93) 1.96 (0.75)	55.852 (1, 432) 74.439 (1, 432)	< 0.001 < 0.001	0.114 0.147

Except for gender identity and completed level of education [N (%)], this table presents averages (and standard deviations) for each group. The p-values (*α* = 0.05), F scores or χ^2^ values, and effect sizes (η^2^ or Cramer’s V) were obtained using one-way ANOVAs for age, years of musical training, and POMS (subscales: depression and tension-anxiety) and chi-square tests for gender and completed level of education. The number of participants for musical training is unequal due to some participants not having received any formal musical training (the question did not appear for them). POMS = Profile of Mood State.

### 3.2 Listening habits for background music

#### 3.2.1 Hours per week spent listening to music (as primary and secondary activity)

Two separate comparisons of medians between groups were conducted using Mann–Whitney U tests. The number of hours per week spent listening to music as a primary activity was significantly (*U* = 16,209, *z* = −2.108, *p* = 0.035) higher in the neurotypical group (*Median* = 3 h, *SD* = 9) compared to the ADHD-screened group (*Median* = 2 h, *SD* = 4). However, these two groups were similar in terms of the number of hours spent listening to music per week as a secondary activity (neurotypical *Median* = 10, *SD* = 13; ADHD-screened *Median* = 10, *SD* = 15; *U* = 18,291, *z* = −0.304, *p* = 0.761).

#### 3.2.2 Comparisons of background music listening between groups (neurotypical and ADHD-screened) and types of activities (more or less cognitive)

The average amount of BM listening (measured using Likert scales ranging from 1 = never to 7 = very often) during more and less cognitive activities was compared between activity type and groups (see Table 4 for statistical indices) using repeated measures ANOVA, with activity type as a within-subject factor and group (neurotypical vs. ADHD-screened) as a between-subjects factor. The average amount of music listening during the four less cognitive activities (*M* = 5.51, *SD* = 1.28) was significantly greater than during the seven more cognitive activities (*M* = 3.72, *SD* = 1.49) [*F*(1, 432) = 420.246, *p* < 0.001, *ƞ^2^_partial_* = 0.493]. The group effect was not significant [*F*(1, 432) = 2.437, *p* = 0.119, *ƞ^2^_partial_* = 0.006], nor was the interaction between the type of activity and the group [*F*(1, 432) = 0.869, *p* = 0.352, *ƞ^2^_partial_* = 0.002].

**Table 4:** Repeated measures ANOVA results for music listening based on activity type and group.

Comparisons	*F*(1, 432)	*p-value*	partial ƞ^2^	M (SD)
Activity type (more vs. less cognitive)	420.246	< 0.001	0.493	
Group (neurotypical vs. ADHD-screened)	2.437	0.119	0.006	
Interaction (activity * group)	0.869	0.352	0.002	
Mean – less cognitive				5.51 (1.28)
Mean – more cognitive				3.72 (1.49)

The scores were collected using a Likert scale ranging from 1 (never) to 7 (very often). *α* = 0.05.

##### 3.2.2.1 Average background music listening for more cognitive activities

The amount of BM listening during each of the more cognitive individual activities was analyzed separately using ANOVAs with a factor (group) with two levels (neurotypical and ADHD-screened). The results indicate that during studying, the ADHD-screened group reported significantly more BM listening compared to the neurotypical group [*F*(1, 432) = 6.49, *p* = 0.011, *η^2^* = 0.015], as detailed in Table 5. In contrast, average BM listening for the six other more cognitive activities, as well as the average of the seven more cognitive activities together, did not differ between groups (neurotypical and ADHD-screened). Since all correlations between potential confounding variables (i.e., years of musical training, scores on POMS subscales) and average BM listening during studying for both groups were non-significant, musical training and POMS scores were not considered in further analyses to explain the significant difference between groups during the more cognitive activity “studying.”

**Table 5:** One-way ANOVA results comparing background music listening amount for more cognitive activities for neurotypical and ADHD-screened participants.

	Neurotypical (*n* = 316)	ADHD-screened (*n* = 118)	*F*(1, 432)	*p-value*	η^2^
While studying While memorizing For problem-solving or calculations While reading While writing While learning (e.g., a new language) For engaging in logic puzzles (e.g., Sudoku)	4.21 (1.97) 3.13 (1.89) 3.65 (1.95) 3.33 (1.89) 3.92 (1.83) 3.01 (1.89) 4.32 (1.96)	4.75 (1.95) 3.36 (2.03) 3.89 (2.14) 3.56 (2.13) 4.25 (2.06) 3.03 (2.13) 4.49 (2.04)	6.49 1.24 1.21 1.16 2.71 0.004 0.647	0.011 0.267 0.271 0.283 0.100 0.952 0.421	0.015 0.003 0.003 0.003 0.006 0.000 0.001
Mean of the seven more cognitive activities	3.65 (1.46)	3.90 (1.58)	2.437	0.119	0.006

The scores were collected using a Likert scale ranging from 1 (never) to 7 (very often). *α* = 0.05.

##### 3.2.2.2 Average background music listening for less cognitive activities

The amount of BM listening during each of the individual less cognitive activities was analyzed separately using ANOVAs with a factor (group) with two levels (neurotypical and ADHD-screened). The means (and standard deviations) of Likert scale ratings for BM listening during less cognitive activities are presented in Table 6. Average BM listening during commuting/public transportation did not differ between the groups (neurotypical and ADHD-screened) [*F*(1, 432) = 2.58, *p* = 0.109, *η^2^* = 0.006]. However, average BM listening “while engaging in sports” differed significantly between the groups [*F*(1, 432) = 9.54, *p* = 0.002, *η^2^* = 0.022]. Indeed, the ADHD-screened group listens significantly more to BM “while engaging in sports” than the neurotypical group. Since correlations between potential confounding variables (musical training and POMS scales scores) and average BM listening during sports (for each group) were non-significant, these confounding variables were not considered in further analyses to explain the significantly higher use of BM during sports in ADHD-screened participants.

**Table 6:** One-way ANOVA results comparing background music listening amount for less cognitive activities for neurotypical and ADHD-screened participants.

	Neurotypical (*n* = 316)	ADHD-screened (*n* = 118)	*F*(1,432)	*p-value*	η^2^
While cleaning During commuting/public transportation While cooking at home While engaging in sports	5.44 (1.57) 5.65 (1.53) 5.08 (1.69) 5.41 (1.62)	5.92 (1.49) 5.91 (1.44) 5.49 (1.59) 5.94 (1.48)	8.011 2.583 5.227 9.537	0.005 0.109 0.023 0.002	0.018 0.006 0.012 0.022
Mean of the four less cognitive activities	5.40 (1.27)	5.81 (1.26)	9.271	0.002	0.021

The scores were collected using a Likert scale ranging from 1 (never) to 7 (very often). Standard deviations are in parenthesis after every average score. α = 0.05.

However, there was a significant correlation between the less cognitive activities “while cleaning” (*r* = 0.151, *p* = 0.002, *r^2^* = 0.023), “while cooking at home” (*r* = 0.142, *p* = 0.003, *r^2^* = 0.020), the combined average of the four less cognitive activities, and the tension-anxiety subscale scores of the POMS. Therefore, three multiple regressions were conducted to examine the influence of tension-anxiety POMS subscale scores and group on BM listening during these three less cognitive activities, i.e., “cleaning,” “cooking at home,” and the average of the four less cognitive activities. For “cleaning,” the predictive model was significant [*F*(2,431) = 6.588, *R^2^* = 0.025, *p* = 0.002], with the tension-anxiety subscale as a significant positive predictor (*β* = 0.116, *t* = 2.256, *p* = 0.025), but no significant relationship with group (*β* = 0.091, *t* = 1.762, *p* = 0.079). For “cooking at home,” the predictive model was also significant [*F*(2,431) = 5.225, *R^2^* = 0.019, *p* = 0.006], with the tension-anxiety subscale as a significant positive predictor (*β* = 0.117, *t* = 2.274, *p* = 0.023) and, again, no significant relationship for the group predictor (*β* = 0.064, *t* = 1.250, *p* = 0.212). However, for the average of the four less cognitive activities, the predictive model was significant [*F*(2,431) = 6.652, *R^2^* = 0.025, *p* = 0.001], with the tension-anxiety subscale (*β* = 0.102, *t* = 1.992, *p* = 0.047) and the group (*β* = 0.106, *t* = 2.058, *p* = 0.040) as significant positive predictors. In summary, the POMS tension-anxiety subscale score is a significant predictor of BM listening during “cleaning” and “cooking at home,” while the group appears to be a more important predictor of music listening for average of the four less cognitive activities.

In summary, the ADHD-screened group reported listening to BM significantly more often than the neurotypical group while studying and, engaging in sports, as well as for the average listening for the four less cognitive activities. However, the tension-anxiety POMS subscale scores better explain the difference in average BM listening habits while cleaning and cooking at home than being neurotypical or ADHD-screened.

#### 3.2.3 Characteristics of background music listening during more cognitive and less cognitive activities

Table 7 presents participants’ preferences in terms of BM characteristics (i.e., relaxing vs. stimulating, with vs. without lyrics, familiar vs. unfamiliar, and self-chosen vs. not chosen) during more cognitive and less cognitive activities. Participants were asked to choose between each of these dichotomic options for both types of activities. During more cognitive activities, both groups seem to prefer listening to relaxing music (neurotypical = 57.3%; ADHD-screened = 44.1%), music without lyrics (neurotypical = 39.2%; ADHD-screened = 46.6%), familiar music (neurotypical = 53.5%; ADHD-screened = 42.4%), and music chosen by themselves (neurotypical = 51.3%; ADHD-screened = 43.2%). Still concerning more cognitive activities, a significantly higher frequency of neurotypical participants than screened with ADHD participants reported preferring relaxing music [*χ^2^* (1, *N* = 434) = 6.031, *p* = 0.014] and familiar music [*χ^2^* (1, *N* = 434) = 4.241, *p* = 0.039], while a significantly higher frequency of participants screened with ADHD than neurotypicals prefer to listen to stimulating music [*χ^2^* (1, *N* = 434) = 5.297, *p* = 0.021]. Groups were equivalent on other preferred BM characteristics (with or without lyrics, unfamiliar, chosen or not by the respondent).

**Table 7:** χ^2^ comparisons of preferred musical characteristics during more and less cognitive activities between groups (neurotypical, ADHD-screened).

		Neurotypical (*n* = 316)		ADHD-screened (*n* = 118)				
Preferred musical characteristics		*n*	%	*n*	%	*χ^2^* (*1, 434*)	*p-value*	Cramer’s V
More cognitive activities	Relaxing Stimulating No preference Does not apply	181 37 84 14	57.3 11.7 26.6 4.4	52 24 35 7	44.1 20.3 29.7 5.9	6.031 5.297 0.409 0.421	0.014 0.021 0.522 0.517	0.118 0.110 0.031 0.031
	Without lyrics With lyrics No preference Does not apply	124 78 96 18	39.2 24.7 30.4 5.7	55 25 29 9	46.6 21.2 24.6 7.6	1.926 0.581 1.411 0.549	0.165 0.446 0.235 0.459	0.067 0.037 0.057 0.036
	Familiar Unfamiliar No preference Does not apply	169 45 91 11	53.5 14.2 28.8 3.5	50 21 40 7	42.4 17.8 33.9 5.9	4.241 0.843 1.061 1.299	0.039 0.359 0.303 0.254	0.099 0.044 0.049 0.055
	Chosen by you Choice does not matter to me No preference Does not apply	162 78 61 15	51.3 24.7 19.3 4.7	51 32 29 6	43.2 27.1 24.6 5.1	2.225 0.269 1.453 0.021	0.136 0.604 0.228 0.884	0.072 0.025 0.058 0.007
Less cognitive activities	Relaxing Stimulating No preference Does not apply	93 132 84 7	29.4 41.8 26.6 2.2	22 73 19 4	18.6 61.9 16.1 3.4	5.132 13.916 5.214 0.480	0.023 < 0.001 0.022 0.488	0.109 0.179 0.110 0.033
	Without lyrics With lyrics No preference Does not apply	40 178 89 9	12.7 56.3 28.2 2.8	11 75 28 4	9.3 63.6 23.7 3.4	0.922 1.847 0.859 0.087	0.337 0.174 0.354 0.768	0.046 0.065 0.044 0.014
	Familiar Unfamiliar No preference Does not apply	188 18 105 5	59.5 5.7 33.2 1.6	76 11 30 1	64.4 9.3 25.4 0.8	0.870 1.811 2.442 0.340	0.351 0.178 0.118 0.560	0.045 0.065 0.075 0.028
	Chosen by you Choice does not matter to me No preference Does not apply	162 67 84 3	51.3 21.2 26.6 0.9	66 21 26 5	55.9 17.8 22.0 4.2	0.750 0.617 0.939 5.133	0.386 0.432 0.332 0.023	0.042 0.038 0.047 0.109

This table presents the N and frequencies of participants’ preferred BM characteristics (i.e., relaxing vs stimulating, with vs without lyrics, familiar vs unfamiliar, and chosen by the respondent vs not chosen). *P*-values, χ^2^ scores, df and effect sizes (Cramer’s V) were obtained using Chi-square independence tests. Given the very small sample of people who selected “does not apply,” these comparisons should be interpreted with caution. *α* = 0.05.

Regarding preferred characteristics during less cognitive activities, both groups seem to prefer listening to stimulating music (neurotypical = 41.8%; ADHD-screened = 61.9%), music with lyrics (neurotypical = 56.3%; ADHD-screened = 63.6%), familiar music (neurotypical = 59.5%; ADHD-screened = 64.4%), and music chosen by themselves (neurotypical = 51.3%; ADHD-screened = 55.9%). Moreover, for this type of activity, a significantly higher frequency of neurotypical adults than those screened with ADHD report preferring to listen to relaxing music [*χ^2^* (1, *N* = 434) = 5.132, *p* = 0.023] or have no preference (relaxing and stimulating) [*χ^2^* (1, *N* = 434) = 5.214, *p* = 0.022], while a significantly higher frequency of adults screened with ADHD than neurotypicals prefer to listen to stimulating music [*χ^2^* (1, *N* = 434) = 13.916, *p* = < 0.001] during less cognitive activities. Other comparisons (with or without lyrics, familiar or unfamiliar, chosen or not by the respondent) between groups regarding musical characteristics for less cognitive activities were not significant.

#### 3.2.4 Music styles

The neurotypical group reported preferring the following music styles: pop (44.9%), classical or opera (34.8%), and jazz or blues (27.2%) during more cognitive activities. The ADHD-screened group reported preferring classical or opera (36.4%), pop (32.2%), and alternative or indie (25.4%). Following a Chi-square independence test, a significant difference was found for one of the 21 musical styles; participants in the neurotypical group are more likely to prefer listening to pop music during cognitive activities than the ADHD-screened group [*χ^2^* (1, *N* = 434) = 5.739, *p* = 0.017, *V* = 0.115]. However, when using Bonferroni corrected alpha (*α* = 0.05 divided by 21 comparisons results in *α* = 0.002), this distinction was no longer significant.

During less cognitive activities, the neurotypical group seems to prefer listening to pop (57.9%), dance/techno/electronic (34.5%), and popular songs from their culture (28.8%). The ADHD-screened group, on the other hand, prefers listening to pop (53.4%), rap or hip-hop (33.9%), and popular songs from their culture (30.5%) during less cognitive activities. Following a Chi-square independence test, a significant difference was found for one of the 21 musical styles. Neurotypical participants were more likely to report listening to world music during less cognitive activities than ADHD-screened participants [*χ^2^* (1, *N* = 434) = 5.734, *p* = 0.017, *V* = 0.115]. However, again, using Bonferroni correction (*α* = 0.05 divided by 21 comparisons results in *α* = 0.002), this distinction was no longer significant.

### 3.3 Subjective effects of background music on cognitive and emotional functioning

#### 3.3.1 Exploratory factor analysis

An exploratory factor analysis was conducted to investigate the structure of the data derived from 27 statements (see Supplementary Table S2) regarding the subjective effect of BM during more cognitive activities. Respondents were required to rate their degree of agreement on a Likert scale from 1 (completely disagree) to 7 (completely agree). To determine the number of factors, both the scree plot and parallel analysis were used. Two factors were extracted, with eigenvalues of 6.9 and 5.7, explaining 50.5 and 11.2% of the total variance, respectively (cumulative = 61.7%). Each factor was interpreted based on the statements strongly associated with it. The first factor was primarily influenced by nine statements (see Table 8). Those statements were associated with the effect of music on cognitive functioning, such as benefits to concentration. The second factor (six statements) was characterized by strong loadings on statements associated with the effects of music on emotional functioning, such as mood modulation (see Table 8). A variable loading matrix was generated to display the associations between each statement and each factor. A Bartlett test of sphericity revealed that the observed correlation structure was significantly different from that expected by chance (*χ^2^* = 4498.03, *p* < 0.001). Eight statements were removed from the scale, as they created a single factor grouping all inverted items from the questionnaire, regardless of the concepts they were measuring. Four additional items were removed due to either low saturation or cross-loading, which affected the clear definition of factors for a sample size of over 300 participants (MacCallum et al., 1999). Saturation ranged from 0.66 to 0.89 for the cognitive functioning factor and from 0.57 to 0.88 for the emotional functioning factor. A significant correlation (*r* = 0.580, *p* < 0.001, *r^2^* = 0.336) between both factors was present, justifying the use of the Oblimin with Kaiser normalization rotation method and confirming that the extracted factors indeed concern cognitive and emotional functioning. Cronbach’s alphas for the factors demonstrate good scale reliability; 0.94 for the cognitive functioning factor and 0.89 for emotional functioning factor. The means and standard deviations of each item are reported in Supplementary Appendix C.

**Table 8:** Factor 1 and 2 saturation for each item (ranked from highest to lowest).

	Factor loadings	
	1	2
Factor 1: cognitive functioning		
My concentration is enhanced thanks to background music. Background music improves my concentration during cognitive activities. My performance is better when I engage in cognitive activities with music. Background music helps me memorize new information. Background music positively influences my performance on cognitive tasks. Background music helps to make me more alert. Background music allows me to concentrate better. Background music acts as a good stimulant for performing cognitive activities. Background music heightens my senses.	**0.89** **0.87** **0.82** **0.80** **0.79** **0.71** **0.70** **0.67** **0.66**	−0.06 0.00 0.02 −0.14 0.03 0.003 0.12 0.15 0.18
Factor 2: emotional functioning factor		
Background music improves my mood. Background music makes me happy. Background music brings me a sense of joy. Background music makes my mood less negative. Background music reduces my stress. Background music reduces my boredom feelings.	−0.07 −0.05 −0.05 0.07 0.18 0.09	**0.88** **0.88** **0.85** **0.71** **0.62** **0.57**

*N* = 434. This table presents the statements belonging to the cognitive functioning factor and the emotional functioning factor. Responses to these items were made using a Likert scale ranging from 1 (total disagreement) to 7 (total agreement). Scree plot and parallel analysis were used to determine the number of factors (Oblimin with Kaiser normalization rotation method). Factor loadings above 0.50 are in bold.

#### 3.3.2 Comparison of the average perceived effects of BM listening on cognitive and emotional functioning between neurotypical and ADHD-screened groups

A one-way ANOVA revealed no significant differences between the neurotypical group and the ADHD-screened group on their average scores for statements related to BM impacts on cognitive functioning [*F*(1, 432) = 0.614, *p* = 0.434, *η^2^* = 0.001] or emotional functioning [*F*(1, 432) = 1.320, *p* = 0.251, *η^2^* = 0.003]. Means and standard deviations are reported in Table 9. The individual items forming the cognitive and emotional factors did not significantly differ either between groups (see Supplementary Appendix C). This comparison helped confirm that the non-significant results between groups for the two factors were not influenced by the fact that the EFA was conducted on the total sample.

**Table 9:** Average subjective effects of background music on cognitive functioning and emotional functioning during more cognitive activities for each group (neurotypical, ADHD-screened).

	Neurotypical (*n* = 316)	ADHD-screened (*n* = 118)
Cognitive functioning	4.79 (1.33)	4.89 (1.15)
Emotional functioning	5.72 (0.96)	5.60 (1.00)

The scores were collected using a Likert scale from 1 (completely disagree) to 7 (completely agree). Standard deviations are in parenthesis after every average score.

## 4 Discussion

The primary aim of this study was to compare background music listening habits during more and less cognitive activities between neurotypical young adults and those screened with ADHD. The secondary objective was to compare, within and between these two groups, the subjective effects of BM on two anticipated factors: cognitive and emotional functioning, with a focus on more cognitive activities. We did not form any hypotheses for the first objective (comparison of listening habits between groups). Although two factors were identified as anticipated, the absence of significant differences between groups for both factors suggests that the subjective effect of background music on cognitive and emotional functioning is similar, regardless of neurotypical or ADHD-screened profile. However, significant differences in listening habits were observed between the neurotypical group and the ADHD-screened group.

### 4.1 Listening habits differ between neurotypicals and ADHD-screened participants

The ADHD-screened group demonstrates a significantly higher preference for listening to BM in certain specific situations, such as when studying, while engaging in sports, as well as for the combined average of the four less cognitively demanding activities, compared to the neurotypical group. Our results also reveal that adults screened with ADHD have a significantly stronger preference for stimulating music compared to neurotypicals, both during more and less cognitively demanding activities. These findings suggest some differences between the two groups in their propensity to use BM, possibly related to the complexity of the activity, the availability of attentional resources, and the need for arousal (Goltz and Sadakata, 2021; Kiss and Linnell, 2023; Kiss and Linnell, 2024; Mendes et al., 2021).

These results can partly be interpreted in light of the Cognitive Capacity Hypothesis (CCH; Kahneman, 1973), which posits that attentional resources are limited and must be shared among different activities. For example, a primary activity, whether more or less cognitive, must optimize the distribution of available attention with the secondary task (music listening) (Chou, 2010; Kotsopoulou and Hallam, 2010). Although it is difficult to precisely determine the level of attentional resources required by a task for each individual (Goltz and Sadakata, 2021), according to the CCH, activities that require higher cognitive load require more attentional resources (Chou, 2010; Kahneman, 1973; Kang and Lakshmanan, 2017; Kotsopoulou and Hallam, 2010). This aligns with the results of a recent literature review, where “performance in difficult tasks was significantly poorer than performance in easy tasks” in the presence of BM (Cheah et al., 2022). Thus, considering the results of this research, an individual screened for ADHD, who may already have reduced attentional reserves compared to neurotypical individuals, might experience excessive cognitive load when listening to BM during a cognitively complex activity, thus favoring BM listening during less cognitive (or complex) activities. This is supported by the work of Weyandt et al. (2017), which shows that adults with ADHD may have altered neuropsychological functions (e.g., in working memory, sustained attention and executive functions) (Butzbach et al., 2019; Onandia-Hinchado et al., 2021), making them more vulnerable to cognitive overload during demanding tasks. Moreover, BM can negatively affect performance on complex cognitive tasks by increasing overall cognitive load (Cheah et al., 2022; Schellenberg and Weiss, 2013). Therefore, it might be more beneficial for adults screened with ADHD to limit BM listening to less cognitive activities, where cognitive load is lower and where music can have a soothing effect without interfering with task performance (Batho et al., 2020; Cheah et al., 2022; Rickson, 2006). However, the CCH alone is not sufficient to explain the significantly higher use of BM by the ADHD-screened group during studying, a cognitively demanding task, compared to the neurotypical group.

Another theory relevant for interpreting our results is the Moderate Brain Arousal model (Sikström and Söderlund, 2007). According to this ADHD model, and considering the sensitivity of individuals with ADHD to environmental stimuli, a moderate level of brain arousal in the dopaminergic system can enhance the performance of individuals with ADHD. However, too little or too much arousal can impair performance (Batho et al., 2020; Sikström and Söderlund, 2007; Strauß et al., 2018; Zentall, 1975). Thus, young adults screened with ADHD may seek additional stimulation to maintain their cognitive engagement, especially during activities like studying, where mind wandering (hypo-arousal) can be more common due to its boring and monotonous nature (Alali-Morlevy and Goldfarb, 2023; Cohen, 2011; Unsworth and Robison, 2016). There is indeed an association between mind wandering and ADHD symptoms, where preferred music listening could help reduce mind wandering by inducing an optimal brain arousal level (Alali-Morlevy and Goldfarb, 2023).

Furthermore, according to the Mood Arousal Theory, music can lead to enhanced performance in various activities, due to its recognized ability to elicit pleasant activation (Thompson et al., 2001). However, music can also be perceived as unpleasant in certain contexts (e.g., by creating a sensation of overstimulation), thus leading to decreased performance (Hunter and Schellenberg, 2010; Juslin et al., 2010; Lonsdale and North, 2011; Papinczak et al., 2015; Schäfer et al., 2013; Schellenberg and Weiss, 2013; Thompson et al., 2001). Pereira et al. (2011) demonstrated that limbic and paralimbic regions involved in emotions, as well as in the reward circuitry of neurotypical individuals, were significantly more active in response to familiar music compared to unfamiliar music. Specifically, brain regions such as the amygdala, orbitofrontal cortex, and nucleus accumbens play a crucial role in the processing of emotions and rewards (Koelsch, 2014). The increased activations associated with familiar music underscores the importance of familiarity in modulating emotional responses and subjective satisfaction. According to the Mood Arousal Theory (Thompson et al., 2001), individuals seek to maintain an optimal level of emotional/mood arousal. Familiar music, by activating reward circuits, evoking positive emotions, and helping reduce arousal to an optimal level of activation, can help achieve this balance (Kiss and Linnell, 2023). This could explain why neurotypical young adults tend to prefer relaxing and familiar music during cognitive activities, as it would adequately address their own arousal needs.

However, familiar music, while often providing more pleasure (Freitas et al., 2018; van den Bosch et al., 2013; Zajonc, 1968), could also lead to either too much activation or boredom, resulting in disengagement and inattention towards the primary task (Malkovsky et al., 2012; Merrill and Niedecken, 2023), and thus be more disruptive for individuals with ADHD. It is therefore possible that they would use less familiar music compared to neurotypicals, which could suboptimally activate their dopaminergic system (reward system), as suggested by Sikström and Söderlund (2007) Moderate Brain Arousal model (Ferreri et al., 2019). In support of this idea, studies have shown that individuals with ADHD have a different sensitivity to stimulation, which can influence their preference for certain types of music. For example, novel and meaningful background stimuli have been associated with increased dopamine in individuals with ADHD (Sethi et al., 2018), thus favoring optimal and moderate arousal and helping them focus on the task, whereas monotonous or familiar stimuli tend to decrease the ability to maintain attention on the task (Sikström and Söderlund, 2007). However, given the gaps in the literature regarding BM listening habits in neurodivergent populations and the fact that the present study is based on self-reported data, these results should be interpreted with caution.

In summary, the results underscore the complexity of the interaction between being neurotypical or screened with ADHD, individual arousal needs, attentional resources, and the use of BM in different situations. BM listeners may adjust their cognitive load to an optimal level based on the resources required for certain types of activities. The results also support the notion that neurotypical individuals and those screened with ADHD may have distinct activation needs to support their cognitive functioning in different contexts. Studies have shown that music can have both positive and negative effects on arousal (Zimmermann et al., 2019) and performance in individuals with ADHD. It can increase or modulate dopamine levels and enhance or decrease concentration (Moderate Brain Arousal model: Sikström and Söderlund, 2007). Our results may suggest that adults screened with ADHD may benefit from a more conscious choice of BM, especially during complex cognitive activities, to minimize its negative impact on their performance. However, further research is needed to better understand the underlying mechanisms of these musical preferences and to develop interventions tailored to the specific needs of adults with ADHD.

### 4.2 Subjective effects of background music on cognitive and emotional functioning

As was expected, the two factors (cognitive and emotional functioning) emerged, providing an in-depth insight into the subjective effects of BM. The first factor, dominated by statements regarding improved concentration and other aspects of cognitive functioning, suggests that BM can have a positive impact on cognitive performance. This corroborates previous research showing that music could act as a cognitive enhancer, promoting concentration and productivity (Cheah et al., 2022; Gonzalez and Aiello, 2019). The second factor highlights the emotional effects of BM, emphasizing its ability to influence individuals’ mood and emotions. This is consistent with the theory that music can act as an emotional regulator, modulating affect and promoting emotional well-being (Chen et al., 2022; de Witte et al., 2020; Hu et al., 2021; Juslin and Västfjäll, 2008; MacDonald, 2013; Papinczak et al., 2015). The emergence of this factor structure shows the robustness of our results, showing that the relationships between statements are not random but follow a meaningful structure. Furthermore, the high Cronbach alpha coefficients for each factor (cognitive and emotional functioning) demonstrate the reliability of the measurement scales used, thereby reinforcing the validity of our results. The absence of significant differences between the neurotypical group and the ADHD-screened group for the mean scores of the two factors could suggest that the effect of BM on cognitive and emotional functioning is perceived as being of the same intensity. This finding highlights the subjective impacts of BM on cognitive and emotional functioning, which can be beneficial to a wide range of individuals.

### 4.3 Strengths and limitations

This study had several strengths. First, we emphasize that this study features a large sample and employs robust tools (e.g., ASRS-5). Additionally, any diagnosis of a neurodevelopmental disorder (except ADHD in the ADHD-screened group), a neurological disorder, or a mental health disorder (depression, anxiety, bipolar disorder) that could mimic ADHD symptoms and therefore affect ADHD screening with the ASRS-5 tool were excluded from our sample, unlike the majority of studies (Baggio et al., 2021; Lewczuk et al., 2024; Ustun et al., 2017), minimizing the influence of such disorders on the results. Second, most group differences showed no significant correlation with average listening habits or potential confounding variables such as years of musical training or scores on the POMS depression and tension-anxiety subscales. Finally, the recruitment of a large number of participants and the efficient overview of BM listening habits and their effects on cognitive and emotional functioning provided in this study constitute major strengths. Indeed, the results emphasize differences in BM listening habits during daily activities between neurotypical and ADHD-screened individuals, depending on their need for activation and their available cognitive resources. We recommend using surveys/questions (such as the one we created for this study) about these different variables before any laboratory experiments studying the effect of music listening on cognition or emotions.

Several limitations were also identified in the study. First, the neurotypical group received significantly more musical training than the ADHD-screened group, suggesting possible differences in the roles played by music in the lives of these groups, which could have impacted the results (although musical training was not correlated with the variables of interest). Furthermore, the interpretation of the results is limited by the fact that only self-reported questions were used in our online study, as opposed to more objective measures. For example, the addition of a “does not apply” option in the survey section measuring BM listening habits would have been beneficial to limit the possible impacts of participants who never listen to BM in certain situations (e.g., the answers of individuals who never study/memorize would not have been included in the average score of their group for this activity). In addition, the specifications of what represent more and less cognitive activities in the survey, which was added to minimize variability in participants’ interpretations of the questions, could also have influenced participants to answer in accordance with what is seen as more appropriate uses of BM during these activities. As such, a social desirability measure could have been added to the survey to evaluate its impact on participants’ answers. Another limitation pertains to the use of a screening tool to separate participants in the neurotypical or ADHD-screened groups. The proportion of individuals who screened positively for ADHD in the final sample is higher than the Canadian prevalence of the disorder (2.9–7.3%) (Hesson and Fowler, 2018; Morkem et al., 2020), but not surprising considering the use of a screening tool rather than a complete diagnostic evaluation. Indeed, screening tools tend to result in more false positives, resulting in a larger proportion of individuals being flagged as possibly having ADHD than would have been observed in the population (Faraone and Biederman, 2005; Polanczyk et al., 2007). However, the specific screening tool used in this study was chosen for its good sensitivity (82.6%) and specificity (85.5%) (Baggio et al., 2021). Additional questions could have been included in the ASRS-5 screening tool to determine, among other things, if ADHD symptoms appeared before the age of 12, as required for an official diagnosis (American Psychiatric Association, 2013). Moreover, although several comorbidities are related with ADHD, the existing literature suggests that untreated ADHD can contribute to the development or worsening of anxiety and depression symptoms (Barkley, 2008; Riboldi et al., 2022; Sahmurova et al., 2022).

Despite the differences observed between groups, it is important to note that the neurotypical and ADHD-screened groups were equivalent in terms of gender identity and age. Additionally, while the frequencies of respondents who had completed a pre-university or undergraduate university program were similar between the two groups, a significantly higher frequency of participants in the neurotypical group had completed a graduate university program. This disparity is consistent with previous research, which shows that ADHD is associated with reduced academic performance, lower levels of education, and differences in educational trajectories (e.g., studying part-time or returning to education later in life) (American Psychiatric Association, 2013; Doray et al., 2012; DuPaul et al., 2021; Henning et al., 2022). However, the proportion of current post-secondary students was equal in both groups, diminishing the probability that this disparity in graduate diploma obtention had a strong impact on the results. Furthermore, the POMS depression and tension-anxiety subscales scores were significantly higher in the ADHD-screened group on average, which aligns with existing literature emphasizing the importance of a holistic approach to care for individuals with ADHD that addresses their emotional and psychological needs (Barkley, 2008; Riboldi et al., 2022; Sahmurova et al., 2022).

### 4.4 Future directions

This study provides significant insights into the music listening habits of young adults. BM listening habits can be influenced by various factors, including the cognitive load of activities and individuals’ activation needs. By recognizing these influences, professionals can develop interventions better tailored to individual needs, using music as a therapeutic or educational tool to promote well-being and cognitive performance. Future research is needed to confirm these results in controlled laboratory settings and to further explore the variables involved. This could include using advanced neuroimaging techniques to examine how different music affects brain activity in various contexts, employing longitudinal studies to assess how listening habits evolve over time, or replicating laboratory studies suggesting favorable impacts of certain musical parameters on cognitive performance (e.g., amplitude modulation; Woods et al., 2024) in clinical or academic settings. Additionally, research should involve younger and older populations and be validated in individuals who have an ADHD diagnosis. To better quantify the difficulty level of each task, a subjective measure using response choices (e.g., easy to difficult), such as the one used in Goltz and Sadakata’s (2021) study, would be relevant. Although musical experience was not a central variable in this study, it could influence participants’ listening habits. Future research could implement validated tools, such as the Goldsmiths Musical Sophistication Index (Müllensiefen et al., 2014), which provides a detailed profile of one’s musical experience and could be related to the listening habits of different groups. Musical proficiency could also be assessed using, for example, the Brief Assessment of Musical Perception (BAMP; Peretz and Vuvan, 2017) and evaluations of rhythmic abilities, such as the Battery for the Assessment of Auditory Sensorimotor and Timing Abilities (BAASTA; Dalla Bella et al., 2017, 2024). Furthermore, other studies could focus on constructing and evaluating playlists (e.g., relaxation music, concentration music) to enable users to optimally utilize their cognitive resources and activation needs. This leads us to wonder if there might be an ideal playlist for different cognitive profiles.

## 5 Conclusion

In conclusion, our results provide new insights into the differences between neurotypical individuals and those screened for ADHD, highlighting variations in activation needs and available cognitive resources. These findings underscore the importance of understanding the diversity of individual experiences within these groups and suggest avenues for future research and clinical interventions.

## Data Availability

The raw data supporting the conclusions of this article will be made available by the authors upon email request.
