# Supplementary material for: Listening habits and subjective effects of background music in young adults with and without ADHD
Source: Front Psychol. 2025 Jan 22;15:1508181. doi: 10.3389/fpsyg.2024.1508181 (PMC11797425; doi:10.3389/fpsyg.2024.1508181)
Supplement: Supplementary file 1 [file Data_Sheet_1.docx]

Supplementary Material

**Appendix A - Portrait of Music Listening Habits Questionnaire**

**Questionnaire: Background Music Listening Habits**

**How many hours per week (on average) do you listen to music as a primary activity?**

Specifically, I listen to music without doing any other activity at the same time.

☐ 0 ☐ 1 ☐ 2 ☐ 3 … ☐ 168

**How many hours per week (on average) do you listen to music as a secondary activity?**

Example: I listen to music while working. In this example, music would be the secondary activity, and working would be the primary activity.

☐ 0 ☐ 1 ☐ 2 ☐ 3 … ☐ 168

The following questions aim to explore your habits regarding background music listening when performing certain activities. Please respond to the statements below by checking the appropriate boxes or writing your answers where applicable.

*Attention.* **MORE cognitive activities** require greater mental effort and concentration than **LESS cognitive activities.**

*Note.* Background music refers to listening to music as a secondary activity while performing a primary task (e.g., listening to music while driving).

Using the scale below, ranging from 1 (Never) to 7 (Very often), indicate how frequently you listen to music during the following MORE cognitive activities.

|  | 1 = Never | 2 | 3 | 4 | 5 | 6 | 7 = Very often |
| --- | --- | --- | --- | --- | --- | --- | --- |
| While studying |  |  |  |  |  |  |  |
| While memorizing |  |  |  |  |  |  |  |
| For problems-solving or calculations |  |  |  |  |  |  |  |
| While reading |  |  |  |  |  |  |  |
| While writing |  |  |  |  |  |  |  |
| While learning (e.g., new language) |  |  |  |  |  |  |  |
| For engaging in logic puzzles (e.g., Sudoku) |  |  |  |  |  |  |  |

Specify which style(s) of music you listen to when performing these MORE cognitive activities:

☐ None ☐ Alternative or Indie ☐ Jazz or Blues ☐ Classical or Opera ☐ Country, Western ☐ Dance, Techno, or Electronic ☐ Latin Music ☐ Ballad ☐ Folk ☐ Gospel ☐ Metal or Punk ☐ Soul ☐ Popular Music from Your Culture ☐ Pop ☐ World Music ☐ Rap or Hip-Hop ☐ Reggae ☐ Traditional Music from Your Culture ☐ Rock ☐ R&B ☐ Music from Films, TV Shows, Video Games ☐ Other: _______

| When you listen to music while performing your MORE cognitive activities, do you prefer the music to be:  *Please respond by checking the appropriate boxes.  ☐ Does not apply ☐ Relaxing ☐ Stimulating ☐ No preference  ☐ Does not apply ☐ Without lyrics ☐ With lyrics ☐ No preference  ☐ Does not apply ☐ Familiar ☐ Unfamiliar ☐ No preference  ☐ Does not apply ☐ Chosen by you ☐ The choice of music does not matter ☐ No preference |
| --- |

Using the scale below, ranging from 1 (Never) to 7 (Very often), indicate how frequently you listen to music during the following LESS cognitive activities.

|  | 1 = Never | 2 | 3 | 4 | 5 | 6 | 7 = Very often |
| --- | --- | --- | --- | --- | --- | --- | --- |
| While cleaning |  |  |  |  |  |  |  |
| During commuting/public transportation |  |  |  |  |  |  |  |
| While cooking at home |  |  |  |  |  |  |  |
| While engaging in sports |  |  |  |  |  |  |  |

Specify which style(s) of music you listen to when performing these LESS cognitive activities:

☐ None ☐ Alternative or Indie ☐ Jazz or Blues ☐ Classical or Opera ☐ Country, Western ☐ Dance, Techno, or Electronic ☐ Latin Music ☐ Ballad ☐ Folk ☐ Gospel ☐ Metal or Punk ☐ Soul ☐ Popular Music from Your Culture ☐ Pop ☐ World Music ☐ Rap or Hip-Hop ☐ Reggae ☐ Traditional Music from Your Culture ☐ Rock ☐ R&B ☐ Music from Films, TV Shows, Video Games ☐ Other: _______

| When you listen to music while performing your LESS cognitive activities, do you prefer the music to be:  *Please respond by checking the appropriate boxes.  ☐ Does not apply ☐ Relaxing ☐ Stimulating ☐ No preference  ☐ Does not apply ☐ Without lyrics ☐ With lyrics ☐ No preference  ☐ Does not apply ☐ Familiar ☐ Unfamiliar ☐ No preference  ☐ Does not apply ☐ Chosen by you ☐ The choice of music does not matter ☐ No preference |
| --- |

**Appendix B - Subjective Effects of Background Music Questionnaire**

**Questionnaire: Background Music Effects on Performance and Emotions**

This part of the survey aims to explore the effect of background music on your performance in daily activities of a "cognitive nature" (e.g., studying, memorizing, reading, writing).

Referring to the scale below, please respond to the following statements by selecting the corresponding number, where 1 = Strongly disagree and 7 = Strongly agree.

Make sure to respond to all statements as accurately as possible.

Note: Background music refers to listening to music as a secondary activity while you perform a primary task (e.g., listening to music while reading).

| 1 = | 2 = | 3 = | 4 = | 5 = | 6 = | 7 = |
| --- | --- | --- | --- | --- | --- | --- |
| Strongly disagree | Disagree | Slightly disagree | Neutral | Slightly agree | Agree | Strongly agree |

| - **1.** | Background music allows me to concentrate better. | 1 2 3 4 5 6 7 |
| --- | --- | --- |
| - 2. | Background music helps me overcome boredom when engaging in cognitive activities. | 1 2 3 4 5 6 7 |
| - **3.** | Background music helps to make me more alert. | 1 2 3 4 5 6 7 |
| - **4.** | Background music brings me a sense of joy. | 1 2 3 4 5 6 7 |
| **5.** | My performance is better when I engage in cognitive activities with music. | 1 2 3 4 5 6 7 |
| **6.** | Background music reduces my stress. | 1 2 3 4 5 6 7 |
| 7. | Background music makes cognitive tasks less boring. | 1 2 3 4 5 6 7 |
| **8.** | Background music improves my mood. | 1 2 3 4 5 6 7 |
| - 9. | - I believe that music interferes with my concentration. | 1 2 3 4 5 6 7 |
| - **10.** | Background music acts as a good stimulant for performing cognitive activities. | 1 2 3 4 5 6 7 |
| - 11. | - Background music reduces my ability to memorize information. | 1 2 3 4 5 6 7 |
| - **12.** | - Background music reduces my boredom feelings. | 1 2 3 4 5 6 7 |
| - 13. | - Background music increases my stress while engaging in cognitive activities. | 1 2 3 4 5 6 7 |
| - 14. | - Background music distracts me from my primary task. | 1 2 3 4 5 6 7 |
| - **15.** | Background music makes me happy. | 1 2 3 4 5 6 7 |
| - 16. | - Background music helps me relax when engaging in cognitive activities. | 1 2 3 4 5 6 7 |
| - 17. | - Background music disrupts my performance on cognitive tasks. | 1 2 3 4 5 6 7 |
| - 18. | - Background music evokes sense of excitation in me. | 1 2 3 4 5 6 7 |
| - **19.** | My concentration is enhanced thanks to background music. | 1 2 3 4 5 6 7 |
| - 20. | - I get stressed when I engage in cognitive activities with background music. | 1 2 3 4 5 6 7 |
| - **21.** | Background music makes my mood less negative. | 1 2 3 4 5 6 7 |
| - **22.** | Background music positively influences my performance on cognitive tasks. | 1 2 3 4 5 6 7 |
| - **23.** | - Background music heightens my senses. | 1 2 3 4 5 6 7 |
| - **24.** | Background music improves my concentration during cognitive activities. | 1 2 3 4 5 6 7 |
| - 25. | - Background music reduces my productivity. | 1 2 3 4 5 6 7 |
| - 26. | Background music takes up too much of my attention while I am doing cognitive activities. | 1 2 3 4 5 6 7 |
| - **27.** | Background music helps me memorize new information. | 1 2 3 4 5 6 7 |

*Note.* Statement numbers in bold indicate those retained after the EFA.

**Appendix C** - **Subjective Effects of Background Music Scores**

Average Group Scores (and Standard Deviations) for Each Item of the Questionnaire about Subjective Effect of Background Music During More Cognitive Activities on Cognitive Functioning and Emotional

|  | Neurotypical  *(n* = 316) | | ADHD-screened (*n* = 118) | *p-value* |
| --- | --- | --- | --- | --- |
| Factor 1: Cognitive Functioning  My concentration is enhanced thanks to background music.  Background music improves my concentration during cognitive activities.  My performance is better when I engage in cognitive activities with music.  Background music helps me memorize new information.  Background music positively influences my performance on cognitive tasks.  Background music helps to make me more alert.  Background music allows me to concentrate better.  Background music acts as a good stimulant for performing cognitive activities.  Background music heightens my senses. | 4.76 (1.61)  4.77 (1.62)  4.89 (1.72)  4.24 (1.76)  4.88 (1.48)  4.49 (1.66)  4.98 (1.63)  5.05 (1.57)  5.02 (1.50) | 4.82 (1.48)  4.97 (1.34)  5.08 (1.45)  4.08 (1.69)  5.06 (1.37)  4.58 (1.56)  5.25 (1.50)  5.19 (1.42)  5.01 (1.42) | | .700  .246  .272  .417  .262  .626  .113  .373  .948 |
| Factor 2: Emotional Functioning Factor  Background music improves my mood.  Background music makes me happy.  Background music brings me a sense of joy.  Background music makes my mood less negative.  Background music reduces my stress.  Background music reduces my boredom feelings. | 5.86 (1.12)  5.80 (1.11)  5.84 (1.17)  5.56 (1.13)  5.68 (1.23)  5.58 (1.35) | 5.78 (1.14)  5.69 (1.16)  5.67 (1.27)  5.39 (1.14)  5.50 (1.41)  5.56 (1.39) | | .522  .335  .191  .176  .201  .916 |

*Note.* This table presents means (and standard deviations). *p-*values (< .05) was obtained using one-way ANOVAs. The scores were collected using a Likert scale from 1 (completely disagree) to 7 (completely agree).
